# AIR-BT, a new badminton-specific incremental easy-to-use test

**DOI:** 10.1371/journal.pone.0257124

**Published:** 2021-09-10

**Authors:** Javier Abián-Vicén, Alfredo Bravo-Sánchez, Pablo Abián

**Affiliations:** 1 Performance and Sport Rehabilitation Laboratory, Faculty of Sport Sciences, University of Castilla-La Mancha, Toledo, Spain; 2 Faculty of Humanities and Social Sciences, Comillas Pontifical University, Madrid, Spain; Universita degli Studi di Milano, ITALY

## Abstract

**Background:**

Badminton is a highly demanding sport characterized by intermittent efforts with energy being provided by both the aerobic and anaerobic systems. To date, no incremental badminton field test has been developed that can be easily used by badminton coaches that requires accessible material for anyone.

**Objectives:**

The purpose of this study was to develop a practical and reliable easy-to use intermittent endurance badminton test for estimating maximal oxygen uptake (VO_2max_).

**Methods:**

Thirty six Spanish badminton players (age: 24.1±10.3 years) performed, on different days of the same week, four incremental protocols to exhaustion in randomized order: a treadmill incremental test, a Yo-Yo intermittent recovery level 1 test (Yo-Yo IR1) and twice the Abian intermittent recovery badminton test (AIR-BT). Oxygen uptake was determined with a breath-by-breath gas analyzer during the incremental treadmill test, and performance in the Yo-yo IR1 and AIR-BT was recorded.

**Results:**

Significant correlations (p<0.001) for Pearson’s product moment coefficient were found between the performance in the AIR-BT and the two non-specific incremental tests (VO_2max_ in the Treadmill Test: r = 0.87, distance in the Yo-Yo IR1: r = 0.86). The regression equation to calculate the VO_2max_ from the AIR-BT time [VO_2max_ = 0.023*(AIR-BT time in seconds)+31.334] showed an adjusted R^2^ of 0.76 and a SEE of 3.34 ml·kg^-1^·min^-1^. There was no significant difference between VO_2max_ obtained by the incremental treadmill test and VO_2max_ calculated using the regression equation (p = 0.644). A paired t-test reported no significant differences between day 1 and day 2 in the AIR-BT time (p = 0.753), the Pearson correlation coefficient between both days was: r = 0.88 (p<0.001) and the intraclass correlation coefficient was 0.875.

**Conclusions:**

The AIR-BT is a valid and reliable on-court test for assessing VO_2max_ in badminton players and may be utilized by coaches and physical trainers for cross-sectional comparison of players and for evaluation of longitudinal changes.

## Introduction

Badminton is one of the most commonly practiced sports in the world and is growing in popularity worldwide [1]. The demands of the sport require a blend of fine technical skills and specific physiological fitness [2, 3]. The temporal structure of badminton matches has been quantified and described in several studies. The timing of events in a badminton match consists of actions of short duration and high intensity (~ 10 s) interspersed by short rest periods (~ 25 s) with an effective playing time of ~ 28%, a shot frequency of over one shot per second and a match duration of ~ 40 min [4–6]. Badminton is a highly demanding sport characterized by intermittent efforts with energy being provided by both the aerobic (60–70%) and anaerobic (30%) systems [5] with a great demand on the alactic anaerobic system and, to a lesser degree, lactic anaerobic metabolism [1, 7]. Badminton players are required to make explosive movements with continuous changes of direction to intercept the shuttlecock before it touches the ground, which generates a high eccentric load mainly in the muscles of the lower limbs [8].

Laboratory testing is commonly used to evaluate physiological training-induced changes and to determine appropriate training intensities. Traditionally, the capacity of an athlete has been evaluated using continuous exercise tests [9]. However, the relevance of these tests to intermittent sports was questioned, so intermittent tests emerged for sports where efforts were not continuous. In this respect, it is worth noting the Yo-Yo intermittent recovery test [10, 11]. This test has rapidly become one of the most extensively studied fitness tests in sports science due to its specificity and practicality for team sports [12]. Individual sports that require intermittent efforts, such as racket sports, have less of a tradition in the development and use of specific tests, although in recent years several authors have validated specific on-court tests for table tennis [13], squash [14–16] and badminton players [1, 17, 18]. To develop a test protocol to monitor the specific fitness of badminton players, it is necessary to consider the nature of the game and identify the most relevant physiological and technical variables that influence badminton performance [17].

Several fatigue-specific incremental field tests developed for badminton players have been reported in the literature [5]. Wonisch et al. [19] developed a continuous incremental test using the modified Conconi Test [20] in which the players had to make three movements in order (two to the front and one to the back of the badminton court), velocity was increased every minute through sound signals from a pacer until the badminton player’s voluntary exhaustion. Among the intermittent incremental field tests, those that use flashing light bulbs to establish the pace of the movements stand out. In addition, these tests usually include uncertainty since the bulbs light up in a randomized order. Chin et al. [1] and Fuchs et al. [18] developed two tests with a 3-min series of movements to six directions on the badminton court (four corners and two midcourt laterals) with a rest between series of 30 s and 45 s respectively and increasing the speed of movement in each series until the voluntary exhaustion of the player. Madsen et al. [17] recently established an incremental on-court test (B-ENDURANCE) on which we have relied for the development of our specific badminton test, derived from the Yo-Yo intermittent recovery test that has been used for a long time in team sports. This test consists of carrying out a series of 8 specific badminton movements on court with 10 s of rest between series. The movements are carried out in a random order towards custom-made sensors positioned at each of the four corners of the singles badminton court and the pace of the movements increases in each of the series until the player’s voluntary exhaustion. The test performance is measured with the total time. Madsen et al. [17] found that performance in the B-ENDURANCE correlated with the elite players’ position in the national ranking.

Despite the large number of badminton players internationally, research on the performance capacity of badminton players in the literature is scarce compared to other sports [5]. In addition, all the tests developed so far require materials that make it difficult for coaches to use [1, 17, 18]. To date, no incremental badminton field test has been developed that can be easily used by badminton coaches that requires accessible material for anyone. Neither has a specific badminton test been developed to allow the indirect calculation of VO_2 max_ in badminton players. Therefore the objectives of this study were: i) to develop an easy-to-use on-court specific intermittent endurance badminton test, ii) to describe badminton players’ physiological parameters from a laboratory treadmill test, iii) to report the validity and the reproducibility of the test and iv) to validate a VO_2 max_ prediction equation for badminton players from their performance in the intermittent endurance badminton test.

## Materials and methods

### Subjects

Thirty-six Spanish badminton players (age: 24.1 ± 10.3 years, height: 171.6 ± 8.5 cm, body mass: 67.1 ± 12.3 kg, body fat: 23.3 ± 8.7%) volunteered to participate in this investigation. Fourteen of them were women and twenty-two were men. All participants had prior badminton experience of at least 5 years and had trained for ~ 2 h · day^-1^, 5 days · week^-1^ (often including a weekly competition) during the previous year. Furthermore, participants had no previous history of cardiopulmonary diseases and were not taking medications for the duration of the study. Before enrolling in the investigation, prospective participants were fully informed of the risks and discomforts associated with the experiments and gave their informed written consent to participate. The study was approved by the Ethics Committee of Clinical Research at the hospital Complex in Toledo in accordance with the latest version of the declaration of Helsinki.

### Study protocol

During the first visit to the laboratory, the weight and height of each participant was measured using a Seca 700 balance with stadiometer (Seca 700, Seca ltd, Germany) with participants wearing light clothing and no shoes. Body composition (fat mass) was assessed by dual emission X-ray absorptiometry (DXA, GE Healthcare, Lunar, Diegem, Belgium) with participants in a supine position as described before [21]. Then subjects were familiarized with the testing procedures employed in the study. The subjects were instructed to arrive at the laboratory in a rested and fully hydrated state, at least 3 h postprandial and they were also asked to abstain from strenuous exercise for at least 24-h prior to each test. Throughout the study period, subjects were instructed to maintain their normal daily activities and food and fluid intake.

All subjects carried out four incremental protocols to exhaustion in a randomized order: a treadmill test (non-specific), a Yo-Yo intermittent recovery level 1 test (Yo-Yo IR1) and twice the Abian intermittent recovery badminton test (AIR-BT). Each test was conducted on different days of the same week under equal standard environmental conditions (temperature ~ 25 ºC, relative humidity ~ 60%) and at the same time of day. The AIR-BT was performed twice on different days to evaluate the reliability of the test, this was done by calculating the relative difference and the coefficient of variation between test and retest. Before each test, all subjects carried out the same warm-up led by the researcher.

### Experimental procedures

#### Laboratory treadmill test

The treadmill incremental test to exhaustion was performed on a motorized HP Cosmos Saturn treadmill (HP Cosmos, Nussdor-Traunstein. Germany). It consisted of an initial three-minute warm-up at 6 km/h. Subjects started the protocol at 8 km/h with a fixed treadmill grade of 1% [22] followed by increases of 0.25 km/h every 15 s (1 km/h every minute). The subjects were encouraged to continue for as long as possible. The test ended with the voluntary exhaustion of the participants.

The VO_2_, carbon dioxide output (VCO_2_) and respiratory exchange rate (RER) were measured using a breath-to breath gas analyzer (CPX/D Med Graphics, St. Paul, MN, USA). The average values of each variable at every 10 s period were used to analyze data and relate them to phase II of the VO_2_ kinetics [23]. Before each test, the analyzer was calibrated with a known gas mixture (12% O_2_ and 5% CO_2_) and the volume sensor was calibrated with a 3-L syringe.

The last complete stage was used to determine VO_2 max_, maximal achieved speed and maximal respiratory exchange ratio (RER_max_). The VO_2 max_ was taken as the highest 30-s mean value attained prior to the subject’s volitional exhaustion. The VO_2 max_ achieved was assessed in the presence or absence of the VO_2_ “plateau” during the protocols [24]. Individual anaerobic threshold (IAT) was obtained from the average of the 3 values recorded for the load immediately before the threshold occurred. The anaerobic threshold was defined by using the following criteria: nonlinear minute ventilation (VE) elevation, VE/VO_2_, VE/VCO_2_ and the inflection point in the RQ curves [25].

#### Yo-Yo intermittent recovery level 1 test

The Yo-Yo IR1 consisted of repeated two 20-m runs at a progressively increased speed controlled by audio bleeps from a tape recorder [10, 12]. Between each running bout the participants had a 10-s rest period, consisting of 2 x 5 m of jogging. When the participant failed to reach the finishing line in time twice, the distance covered was recorded and represented the test result. The test was performed indoor on a 2-m-wide and 20-m-long running lane marked by cones. Another cone placed 5 m behind the finishing line marked the running distance during the active recovery period. The testing procedures for the Yo-Yo IR1 have been described in detail elsewhere [10, 12]. VO_2 max_ from the Yo-Yo IR test results was estimated by the formula proposed by Bangsbo et al. [12]: VO_2 max_ (mL·kg^-1^·min^-1^) = Yo-Yo IR distance (m) × 0.0084 + 36.4.

#### Abian intermittent recovery badminton test

**Fig 1:**
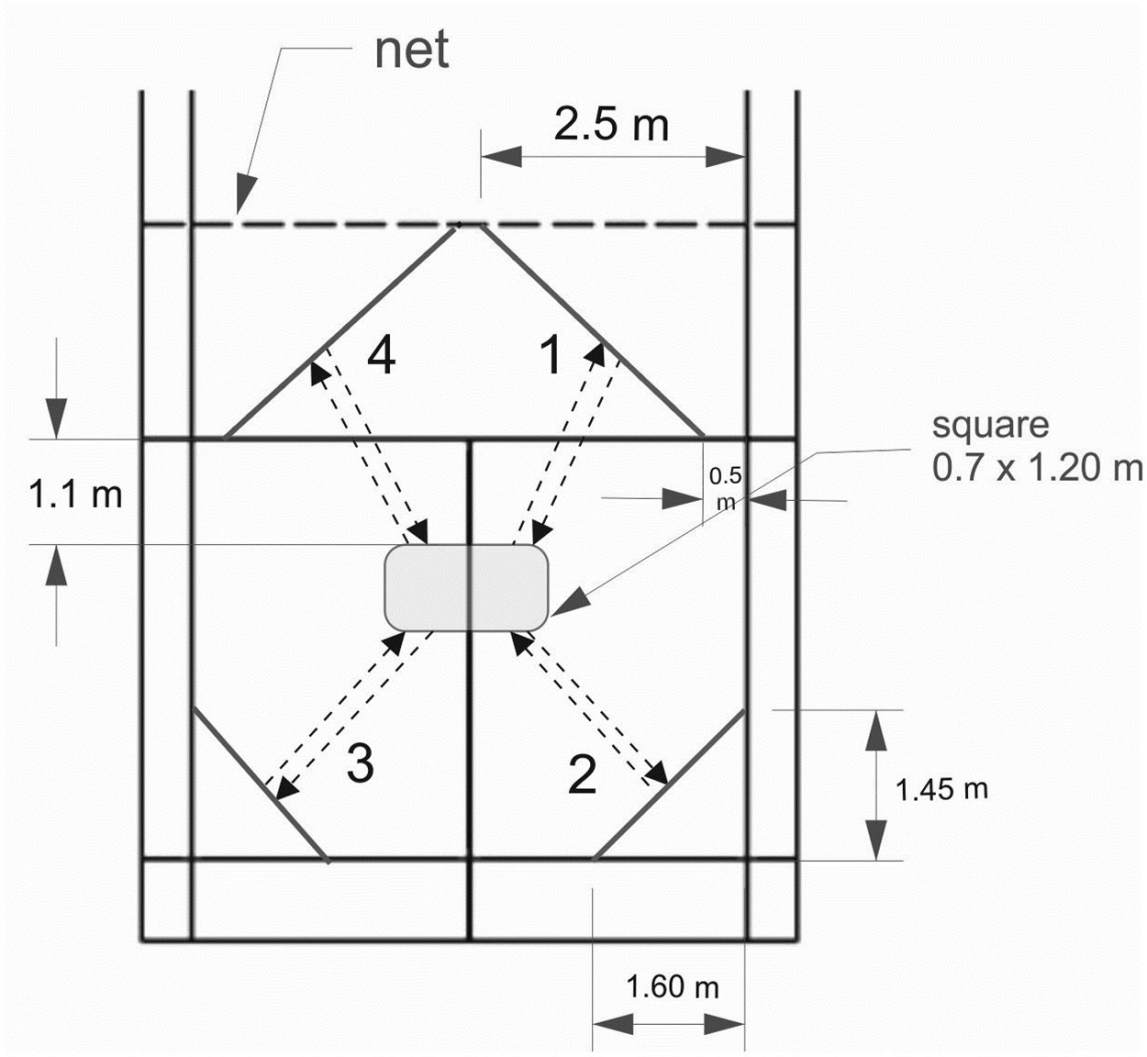
Overview of the AIR-BT marks on the badminton court.

The AIR-BT consisted of 16 levels (level 1–16) made up of different repetitions of a sequence. Each sequence consisted of eight badminton-specific displacements. In each displacement the dominant foot had to cross a line placed at each of the four corners of the singles badminton court returning to the center of the court after performing each of the displacement. Players began by moving from the center to the front right-hand corner of the court and then repeating the cycle by progressing in a clockwise direction through the back right, back left and front left of the court (two rounds in each sequence = eight badminton specific actions). In each movement, the player had to simulate the execution of a badminton stroke and had to return by placing both feet in the rectangle located in the center of the court (Fig 1).

Level 1 and 2 consisted of one sequence, from level 3 to level 7 of three sequences, from level 8 to 12 of four sequences and from level 13 to level 16 of six sequences, with the tempo gradually increasing from each level to the next (Table 1). The participant started a sequence from the center of the badminton court, and time to complete a sequence decreased gradually. Level 1 had a rate of 18 beats/min and the rate increase by 1 beat/min at each level (from 18 beats/min at level 1 to 33 beats/min at level 16). Between each sequence players had an active rest of 10 s. They were requested to simulate a badminton stroke passing the line marked in each direction with their dominant foot. The test was terminated the second time a subject failed to reach the middle position in time with the audio tone within a sequence, and total time and level were recorded as the test results.

**Table 1:** AIR-BT protocol, correspondence between level, time and distance.

level	time (s)	distance (m)	level	time (s)	distance (m)	level	time (s)	distance (m)
1	27	40	8.4	657	840	13.4	1201	1640
2	61	80	9.1	685	880	13.5	1227	1680
3.1	95	120	9.2	713	920	13.6	1253	1720
3.2	129	160	9.3	742	960	14.1	1279	1760
3.3	162	200	9.4	771	1000	14.2	1304	1800
4.1	195	240	10.1	799	1040	14.3	1330	1840
4.2	228	280	10.2	826	1080	14.4	1355	1880
4.3	260	320	10.3	854	1120	14.5	1380	1920
5.1	292	360	10.4	882	1160	14.6	1406	1960
5.2	324	400	11.1	909	1200	15.1	1430	2000
5.3	356	440	11.2	936	1240	15.2	1455	2040
6.1	387	480	11.3	963	1280	15.3	1480	2080
6.2	418	520	11.4	991	1320	15.4	1505	2120
6.3	449	560	12.1	1017	1360	15.5	1530	2160
7.1	479	600	12.2	1044	1400	15.6	1555	2200
7.2	510	640	12.3	1071	1440	16.1	1580	2240
7.3	540	680	12.4	1097	1480	16.2	1604	2280
8.1	569	720	13.1	1123	1520	16.3	1629	2320
8.2	598	760	13.2	1149	1560	16.4	1653	2360
8.3	628	800	13.3	1175	1600	16.5	1678	2400
						16.6	1702	2440

### Statistical analysis

Data analysis was performed using SPSS v 20.0 (SPSS Inc., Chicago, IL). Data are expressed as means ± SD for 36 badminton players. The distribution of datasets was checked using the Kolmogorov-Smirnov test, which demonstrated that all data had a normal distribution (p > 0.05). Descriptive statistics, including means and standard deviations, were calculated for the variables. Pearson correlation coefficients were used to examine the relations between study variables.

Prediction equations for estimating VO_2 max_ were developed by running a simple linear regression analysis with VO_2 max_ calculated in the treadmill incremental test as the dependent variable and the AIR-BT time as the predictor. Adjusted R^2^ and standard error of the estimate (SEE) were used to assess the goodness-of-fit of the prediction model. The validity of the developed equation was evaluated with the Pearson correlation coefficient, the coefficient of determination (R^2^), Student’s t-test, the mean signed difference and the Bland-Altman plot.

The test retest reliability for AIR-BT performance was calculate with day 1 and day 2 trials [26]. In addition, the following criteria of reproducibility in human performance were explored [26, 27]: a) a paired sample t-test was performed to identify the change in the mean, the 95% confidence intervals (CI) and the standard deviation of the difference between the day 1 and the day 2 performance. b) the precision of measurement was determined using the typical percentage error (within-subject variation in percentage). The typical percentage error as a coefficient of variation (CV_TE_) and its 95% CI were calculated using the log-transformed data via the following formula: 100 (e^s^-1), where s is the typical error (standard deviation of the difference between pairs of trials /√2). Logarithmic transformation of the data was performed and used to reduce the possible heteroscedasticity of the raw data [27]. To interpret the CV_TE_ values, the current study used the arbitrary value suggested by Stokes [28] with an analytical goal of 15% or below. In addition, a third aspect was explored: c) assessment of the relative reliability was achieved using an intraclass correlation coefficient (ICC) that indicates the error in measurements as a proportion of the total variance in scores. For all statistical analyses, p<0.05 was accepted as the level of significance.

## Results

All badminton players satisfied the presence of the VO_2_ “plateau” during the last period of the treadmill incremental test to exhaustion, therefore we can consider that all the analyzed tests were maximum. Treadmill tests lasted an average of 8.4 ± 1.8 min and mean maximal running velocity was 16.3 ± 1.9 km·h^-1^. Peak values for heart rate and RER_max_ were 194 ± 12 beats · min^-1^ and 1.17 ± 0.07, respectively. The badminton players showed a VO_2 max_ = 50.2 ± 6.7 ml·kg^-1^·min^-1^, VO_2 IAT_ = 41.5 ± 5.3 ml·kg^-1^·min^-1^ and a velocity at IAT = 13.2 ± 1.7 km·h^-1^.

**Fig 2:**
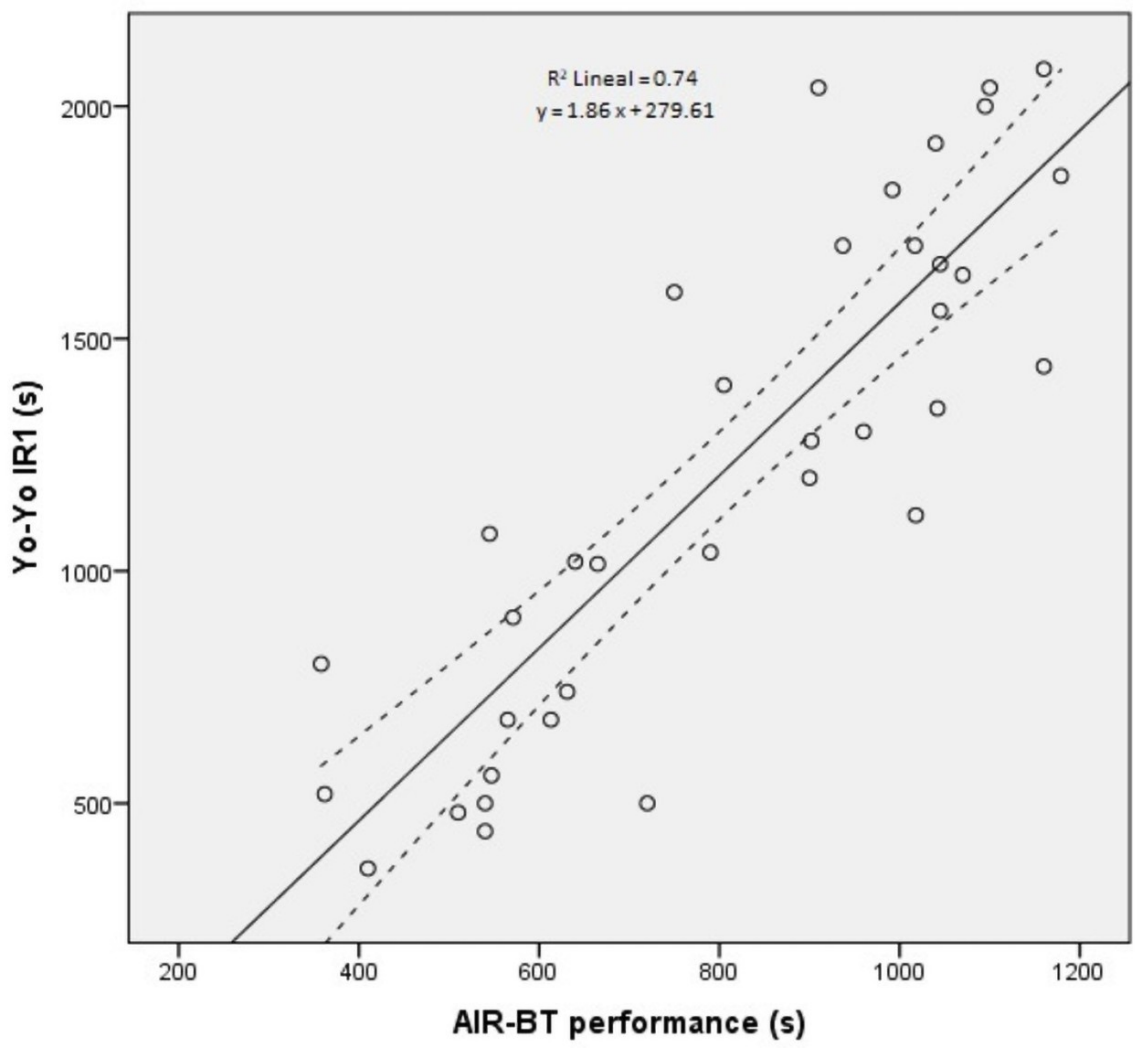
Scatter plot with regression line of the relationship between AIR-BT and Yo-Yo IR1 performance of 36 badminton players.

Performance in the AIR-BT was 809 ± 251 s which corresponds to a level of 9.9 ± 2.4 and a distance covered of 1050 ± 362 m. The distance covered in the Yo-Yo IR1 test was 1226 ± 538 m, which corresponds to a test duration of 10.1 ± 4.3 min, a level of 16.4 ± 1.7 and a calculated VO_2 max_ of 46.7 ± 4.5 ml·kg^-1^·min^-1^. A significant correlation was found between performance in the AIR-BT (time) and Yo-Yo IR1 (distance) (r = 0.86, p<0.001; Fig 2).

**Fig 3:**
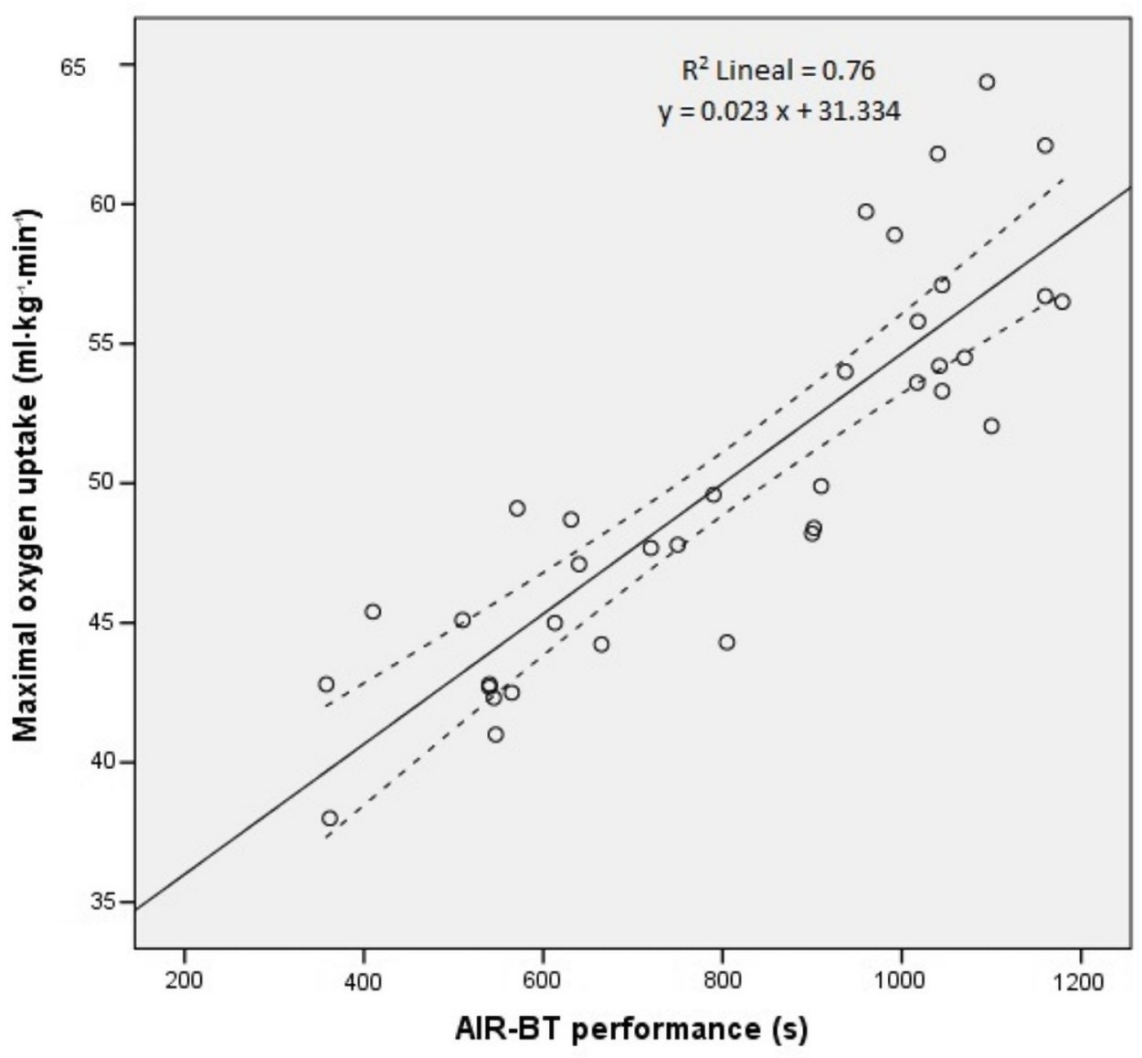
Scatter plot with regression line of the relationship between AIR-BT performance and maximal oxygen uptake from incremental treadmill test of 36 badminton players.

**Fig 4:**
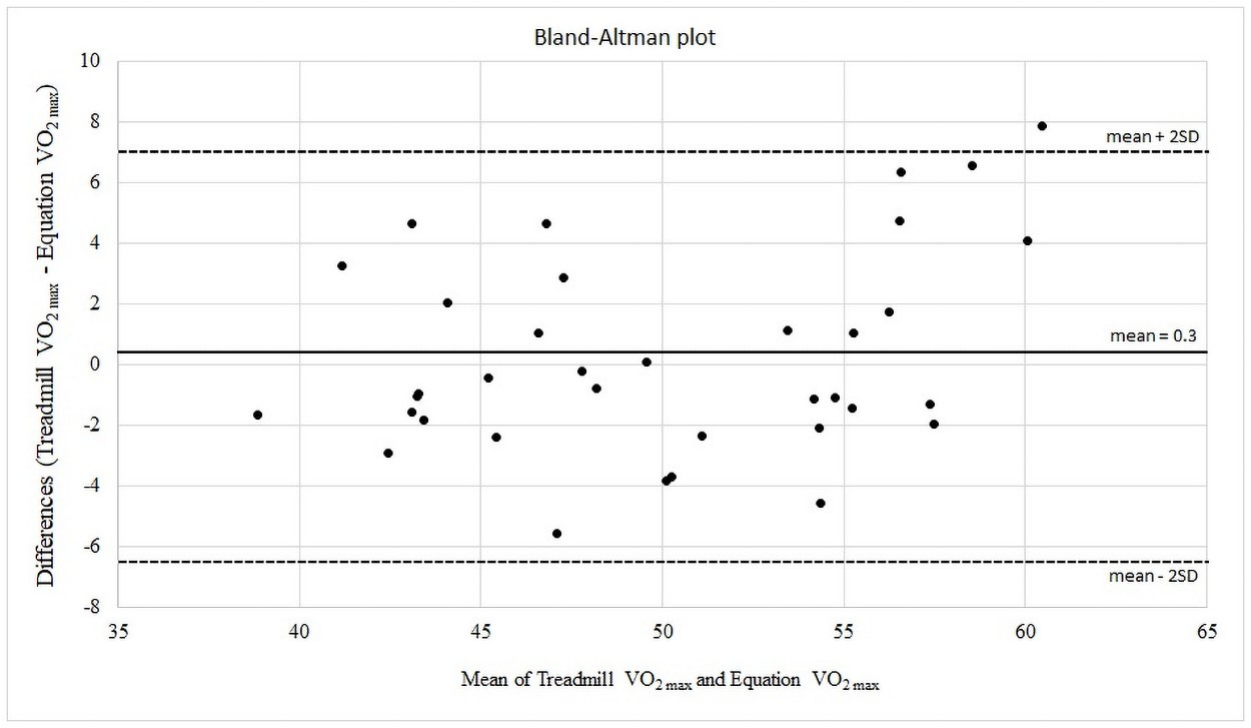
Bland-Altman plot showing the limits of agreement between measured VO_2 max_ (treadmill test) and predicted VO_2 max_ by the equation. The center line represents the mean differences between the two methods, and the other two lines represents two SDs from the mean.

A significant correlation was found between AIR-BT time and VO2 _max_ measured with the incremental treadmill test (r = 0.87, p<0.001). The regression equation [VO_2 max_ = 0.023 * (AIR-BT time in seconds) + 31.334] showed an adjusted R^2^ of 0.76 and a SEE of 3.34 ml·kg^-1^·min^-1^ (Fig 3). The equation for VO_2 max_ was applied to the validation group (n = 36). Estimated VO_2 max_ from equation was 49.9 ± 5.8 ml·kg^-1^·min^-1^, it significantly (r = 0.87, p<0.001) correlated with VO_2max_ by the incremental treadmill test. There was no significant difference between VO_2 max_ obtained by the incremental treadmill test and VO_2 max_ calculated using the regression equation (mean difference = 0.26; 95% CI: from -0.86 to 1.37; p = 0.644). Fig 4 is a Bland-Altman plot illustration of the agreement between measured VO_2 max_ by treadmill incremental test and predicted VO_2 max_ by the equation. Each data point represents the difference between the two methods for each subject.

The paired t-test analyzed reported no significant differences between day 1 and day 2 in the AIR-BT time (Change in the mean = 6.22 s; 95% CI = from -33.6 s to 46.1 s; p = 0.753) nor in the AIR-BT level (Change in the mean = 0.09; 95% CI = from -0.29 to 0.48; p = 0.635) reached by the subjects. The CV_TE_ between days were 11.4% (95% CI = from 9.2% to 15.2%) in the AIR-BT time and 9.2% (95% CI = from 7.4% to 12.1%) in the AIR-BT level. The Pearson correlation coefficients between both days were: r = 0.88 (p < 0.001) in the AIR-BT time and r = 0.88 (p < 0.001) in the AIR-BT level. The ICCs were high in both AIR-BT variables, the AIR-BT time registered an ICC of 0.875 (IC 95% from 0.769 to 0.934) and the AIR-BT level registered an ICC of 0.866 (IC 95% from 0.753 to 0.929).

## Discussion

The current study aimed at validating a new incremental intermittent on-court badminton test (AIR-BT) by investigating its predictive power for VO_2 max_ of the badminton players. The results of this study indicate that the performance in the AIR-BT showed a high correlation with validated and standardized tests for the calculation of VO_2 max_ like the incremental treadmill tests or to assess performance in intermittent sports like the Yo-Yo IR1. Furthermore, test-retest reproducibility was quite high which would indicate that it is an adequate test to observe the evolution of the players during the season. From a practical point of view, the AIR-BT is a specific badminton test to analyze the performance of badminton players, that requires simple instruments to perform. We suggest that the AIR-BT may be utilized by coaches for cross-sectional comparison of players and for evaluation of longitudinal changes.

The VO_2 max_ values recorded from the incremental treadmill test in our study are similar to those obtained in other studies that analyzed racket sports with physiological characteristics very close to badminton, such as squash [14, 15] or tennis [29]. The VO_2 max_ for the Spanish badminton players of this study was lower than reported for elite badminton players (range from 55 to 62 ml·kg^-1^·min^-1^) measured during graded treadmill tests [7, 30–35]. Nevertheless, VO_2 max_ values in the present study are within the values reported for internationally ranked women badminton players by Faude et al. [32] (50.3 ± 4.1 ml·kg^-1^·min^-1^) assessed with a graded treadmill test or the values reported for elite English singles men players by Alcock and Cable [36] (50.6 ± 4.6 ml·kg^-1^·min^-1^) predicted from a multistage 20-meter shuttle-run. We must consider that the sample in the present study was broader and more heterogeneous than what is usually used by studies that analyze the physiological characteristics of badminton players [2, 31, 32, 37]. All the subjects who participated in our study were badminton players who had been training regularly for at least 5 years but their level was heterogeneous, some of them competing nationally and internationally, but others practicing badminton in an amateur way.

Laboratory- based exercise protocols are challenged to assess the specific physiological, metabolic, and technical demands of a specific sport. In common with other racket sports, success in badminton depends on technical, tactical, and motor skills. However, at the highest standard, aerobic fitness is an essential attribute. There are several studies that have developed specific badminton tests to assess player performance [1, 17–19]. All these studies agree on the need to develop specific tests that included badminton-specific movements to assess the physical performance and physiological aspects of the badminton players. This statement is based on the fact that these authors have found a significant correlation between the performance in the endurance badminton-specific tests and the players ranking position and this correlation did not appear with the non-specific tests [1, 17, 18].

The AIR-BT is designed to be performed by badminton players of any level, but it is necessary for players to know the specific footwork technique to perform the movements. It is mainly based on the specific badminton test (B-ENDURANCE) developed by Madsen et al. [17] and on the Yo-Yo IR1 concept. The distance covered in each movement is 5 m (from the center to cross the line and return) and in each sequence of eight badminton-specific actions the player covers a distance of 40 m similar to that proposed in the Yo-Yo IR1 [10, 12]. However, in the AIR-BT, two specific badminton factors were taken into account: i) the change of direction in each movement with its consequent eccentric and concentric action at the muscular level and ii) the simulation of the badminton stroke in each movement. The specific badminton tests developed to date required the use of specific and expensive materials to carry them out [1, 17, 18]. For example, for the B-ENDURANCE [17] the trainer needs custom-made sensors and a computer program to perform the test or in the badminton-specific endurance test developed by Fuchs et al. [18] a computerized board with 6 flashing lights bulbs was necessary to perform the test. However, one of the purposes to be achieved with the AIR-BT was that it could be used by any badminton coach to keep track of their players regardless of the financial resources available to them. Therefore, to use the AIR-BT only the following materials are necessary: 1. A tape measure and a marker pen or a scotch tape to make some marks on the floor (Fig 1), 2. A stopwatch, and 3. An audio that can be downloaded for free at the following link (https://soundcloud.com/user-547665586/abian-interval-recovery-badminton-test-air-bt).

A high correlation was found between the performance in the AIR-BT and the performance in the two non-specific tests measured in the study (incremental treadmill test and Yo-Yo IR1). This reveals that the AIR-BT elicits maximal aerobic responses and it is in line with the metabolic demands of a badminton match where the aerobic system makes a higher contribution (~70%), whilst the anaerobic system makes a lower one (~30%) [7, 32]. These results confirm the validity of the AIR-BT to measure the aerobic capacity of badminton players. The high correlation registered between the time in AIR-BT and the VO_2 max_ of the incremental treadmill test has been used to establish a regression equation that allows us to indirectly calculate the VO_2 max_ from the time recorded in the AIR-BT. Regression equations have been previously used to indirectly calculate VO_2 max_ from continuous tests such as the Cooper’s 12-minute run [38] or the multistage 20-metre shuttle-run [9] and have also been used with somewhat less precision in intermittent tests such as Yo-Yo IR1 and Yo-Yo IR2 [10–12]. The regression equation calculated from AIR-BT has shown very good values of adjusted R^2^ and the SEE. In the application of the regression equation on the validation group, we found a high correlation between the VO_2 max_ calculated directly in the incremental treadmill test and the VO_2 max_ calculated with the regression equation from the AIR-BT and no significant differences were found between both measurements. This indicates that the performance in the AIR-BT is a good indicator of the maximal aerobic response of the players. A possible bias have been found when the VO_2 max_ is calculated with the regression equation in the range between 50 and 55 ml·kg^-1^·min^-1^ where the calculation would underestimate the value of the VO_2 max_ of the players. The VO_2 max_ assessment can allow coaches to compare their players values with other athletes from other sports and also control the evolution of their players’ values longitudinally.

The reliability of the AIR-BT showed no significant differences between the first session and the second session in the performance of the test. Moreover, significant and high correlations were revealed for the AIR-BT time and level (ICC range: 0.89–0.90). The CV_TE_ values were in accordance with other studies that develop sport-specific badminton tests [17, 35]. The test has been carried out with badminton players of different levels, which has made the coefficients of variation somewhat higher. In highly skilled players with a greater stabilization of the technical pattern of movement and a more stable and efficient motor pattern of movement, the reproducibility was higher (~ 3%). It appears that AIR-BT is reproducible, and that coaches and physical trainers may apply the test to evaluate training effects, identifying potentially weak points regarding the player’s physical capacity. Hence, the AIR-BT is suitable in assessing elite badminton players’ physical fitness status.

Some limitations must however be underlined. Firstly, the heterogeneity in the level of the badminton players analyzed that has generated greater variability in the reproducibility of the test. A second main limitation is that the AIR-BT is a specific test only for badminton players and the results of this study can only be extended to badminton players who have mastered the specific footwork technique. This is because the movements that are proposed to developed the AIR-BT require an adequate footwork technique to be reproducible. Finally, the regression equation to indirectly calculate the VO_2 max_ from the performance in the AIR-BT was tested in badminton players with specific characteristics, limiting the ability to generalize the findings to badminton players with different age range or badminton experience.

## Conclusions

In conclusion, this study developed an incremental specific badminton test (AIR-BT) to measure the maximal aerobic response of badminton players. The results of this study demonstrate that the AIR-BT offers a valid and reliable method for assessing VO_2 max_ in badminton players. The performance in the AIR-BT showed a high correlation with validated and standardized tests for the calculation of VO_2 max_ like the incremental treadmill tests or to assess performance in intermittent sports like the Yo-Yo IR1. The regression equation [VO_2 max_ = 0.023 * (AIR-BT time in seconds) + 31.334] allows, by measuring the performance in the AIR-BT, to indirectly calculate the VO_2 max_ of badminton players. Furthermore, test-retest reproducibility was quite high which would indicate that it is an adequate test for observing the evolution of the players during the season.

## Supporting information

S1 Data (XLSX)
